# Importance of Deubiquitination in Macrophage-Mediated Viral Response and Inflammation

**DOI:** 10.3390/ijms21218090

**Published:** 2020-10-29

**Authors:** Roya Rasaei, Neha Sarodaya, Kye-Seong Kim, Suresh Ramakrishna, Seok-Ho Hong

**Affiliations:** 1 Department of Internal Medicine, School of Medicine, Kangwon National University, Chuncheon 24341, Korea; royarasaei93@gmail.com; 2 Graduate School of Biomedical Science and Engineering, Hanyang University, Seoul 04763, Korea; neyha19@gmail.com (N.S.); ks66kim@hanyang.ac.kr (K.-S.K.); 3 College of Medicine, Hanyang University, Seoul 04763, Korea

**Keywords:** macrophages, innate immunity, E3 ligases, DUB inhibitors, RIG-I-like receptor, Toll-like receptor, NOD-like receptor

## Abstract

Ubiquitination and deubiquitination play a fundamental role in the signaling pathways associated with innate and adaptive immune responses. Macrophages are key sentinels for the host defense, triggering antiviral and inflammatory responses against various invading pathogens. Macrophages recognize the genetic material of these pathogens as pathogen-associated molecular patterns (PAMPs) and danger-associated molecular patterns (DAMPs) through the activation of its pattern recognition receptors (PRRs), initiating the cascade of immune signaling, which leads to the production of pro- and anti-inflammatory cytokines that initiates the appropriate immune response. Macrophage-mediated immune response is highly regulated and tightly controlled by the ubiquitin system since its abnormal activation or dysregulation may result in the severe pathogenesis of numerous inflammatory and autoimmune diseases. Deubiquitinating enzymes (DUBs) play a crucial role in reversing the ubiquitination and controlling the magnitude of the immune response. During infection, pathogens manipulate the host defense system by regulating DUBs to obtain nutrients and increase proliferation. Indeed, the regulation of DUBs by small molecule inhibitors has been proposed as an excellent way to control aberrant activation of immune signaling molecules. This review is focused on the complex role of DUBs in macrophage-mediated immune response, exploring the potential use of DUBs as therapeutic targets in autoimmune and inflammatory diseases by virtue of small molecule DUB inhibitors.

## 1. Introduction

Macrophages, first described by Russian zoologist Metchnikoff in 1892, are a key player in innate, as well as adaptive, immunity. Macrophages are ubiquitous cells, derived from embryonic yolk sac or fetal liver precursors during embryonic development, and from bone marrow-derived blood monocytes during adult hematopoiesis [1,2]. Macrophages, a part of the innate immune system, are present throughout the body tissues (e.g., peripheral macrophages in the blood, peritoneal macrophages in the peritoneal cavity, pulmonary macrophages, Kupffer cells [KCs] in the liver, and microglia in the brain) and exhibit great functional diversity [3,4]. They have an essential role in maintaining tumor progression and tissue homeostasis, supporting tissue development, repairing tissue damage, and supporting angiogenesis. As a part of innate immunity, they conserve phagocytic activity and initiate protective inflammatory responses for the recognition and elimination of microbes like bacteria, fungi, and viruses. However, they also exhibit functions associated with T and B cell response as a part of adaptive immunity [5,6].

During viral infection, macrophages are activated, producing an antiviral response and inflammation resulting in pathogen destruction. Macrophage activation is demonstrated by intracellular signaling cascades, which start with the recognition of single- or double-stranded viral RNAs as pathogen-associated molecular patterns (PAMPS) through pattern recognition receptors (PRRs) including toll-like receptors (TLRs); cytoplasmic retinoic acid-inducible gene I (RIG-I)-like receptors (RLRs); and melanoma-differentiation-associated gene 5 (MDA5), also known as Ifih1 or Helicard, and nucleotide-binding oligomerization domain (NOD)-like receptors (NLRs) [7,8,9,10]. PAMP-PRR interaction evokes an antiviral response by producing cytokines such as type I interferons (IFNs), interleukin (IL)-1β, and inducible nitric oxide synthase (iNOS), which causes the virus-infected cell to undergo apoptosis [7,11]. TLRs and RLRs play a central role in warning the host cell against any invading pathogen and to initiate an immune response by both the innate and adaptive immune systems. Recent advances have shown that RLR-mediated signal transduction inducing antiviral immune response is controlled stringently by post-translational modifications (PTMs) including ubiquitination and deubiquitination [12,13].

Another host cell arsenal against infecting microbes is the macrophage inflammatory activation, which is also regulated by ubiquitination and deubiquitination; dysregulated ubiquitination leads to several inflammatory diseases. The inflammasome is a multi-protein oligomer and is responsible for the regulation of inflammatory responses by promoting the maturation and secretion of proinflammatory cytokines from activated macrophages [14]. The formation of inflammasomes is induced by the assembly of both the absent in melanoma 2 (AIM2) receptor and nucleotide-binding oligomerization domain leucine-rich repeat and pyrin domain-containing protein 3 (NLRP3), in response to PAMPs or DAMPs (damage-associated molecular patterns) [15]. This activated NLRs/AIM2 is linked to caspase-1 by an adaptor protein, apoptosis-associated speck-like protein containing a caspase activation and recruitment domain (ASC). Activated caspase-1 releases cytokines and leads ultimately to cell death via pyroptosis. Proinflammatory cytokines (IL)-1β and IL-18, which drive inflammation, are regulated by selective transcription and circulate in nearly every organ in the human body. Dysregulated inflammasome activation and excessive circulating cytokines lead to chronic inflammation and metabolic and autoimmune diseases [16,17,18]. Reversible PTMs like ubiquitination and deubiquitination are, hence, critical for limiting tissue damage and eliciting an adequate immune response.

Ubiquitination is a PTM that regulates most cellular processes including immune regulation. In this process, the carboxy-terminal glycine of ubiquitin (Ub) is covalently attached to a lysine group of the target protein either as a monomer or as a poly-Ub chain. The poly-Ub chain is attached to one of the seven lysine residues, K6, K11, K27, K29, K33, K48, or K63, each of which leads to a distinct outcome for the target protein. For example, K6 and K48 chains mainly target proteins for proteasomal degradation, K11 is involved in endoplasmic reticulum-mediated degradation pathways and control of cell cycle progression, and K29 in regulating the lysosomal degradation of proteins [19]. The roles of K27 and K33 poly-ubiquitination are less understood; however, they have been linked to innate immunity and immune responses [20]. Ubiquitination is sequentially mediated by three enzymes: the ubiquitin-activating enzyme (E1), the ubiquitin-conjugating enzyme (E2), and the ubiquitin ligase (E3). The roles of ubiquitination in inflammasome assembly and activation and macrophage-mediated antiviral response have been extensively studied. Ubiquitination is a reversible process and is executed by deubiquitinating enzymes (DUBs). Families of proteases that cleave the ubiquitin molecule from the ubiquitin-conjugated proteins are called DUBs, and the process is known as deubiquitination. DUBs also maintain the ubiquitin system equilibrium by releasing newly synthesized free Ub precursors and removing ubiquitin from the proteins destined for degradation [21]. However, many pathogens also attack host cell ubiquitin and ubiquitin-like pathways for their survival and proliferation [21,22,23]. Therefore, it is important to provide a better understanding regarding the DUBs regulating macrophages for its antiviral and inflammatory response against invading pathogens and certain inhibitors of ubiquitin machinery that have paved the way as an attractive therapeutic approach for aberrant immune response.

## 2. Ubiquitination and Deubiquitination in the RLR Pathway

### 2.1. K63-Linked Ubiquitination: RIG-I Activation and Cytosolic Warriors

**Figure 1:**
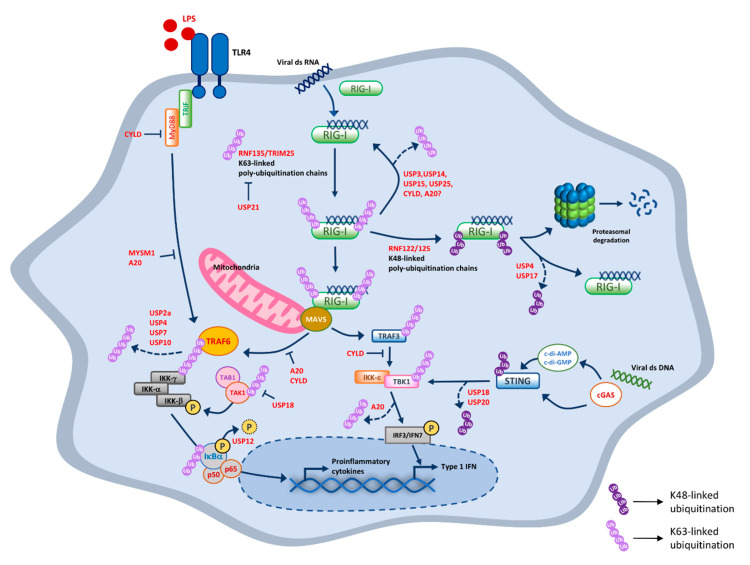
Regulation of the antiviral immune responses by ubiquitination in macrophages. After recognition of cytoplasmic viral RNA species, TLRs and RLRs signals through the downstream adaptors MyD88/TRIF and MAVS, respectively. The MyD88/TRIF adaptor then propagate TLR signal to TRAF6 and NF-κB (p50 and p65) activation. K63-linked polyubiquitination recruits the TAK1/TAB1 complex, which leads to phosphorylation of IκBa. Degradation of IκBα by phosphorylation releases the p50 and P65, leading to induction of proinflammatory cytokines. RLRs signals via the mitochondrial adaptor MAVS are strictly regulated by K63- and K48-linked polyubiquitination. RIG-I is activated by K63-linked polyubiquitination mediated by TRIM25, which is inhibited by USP21. On the other hands, RNF125-mediated K48-linked polyubiquitination of RIG-I induces its degradation by the proteasome, which can be inhibited by USP4 and USP17. The cytoplasmic viral DNA can be recognized by cGAS, which activates STING via synthesizing the second messenger (c-di-GMP and c-di-AMP) and K48-lined ubiquitination. Activated STING facilitates type I IFN gene expression. Dark purple circles indicated K48-linked ubiquitination and light purple circles indicate K63-linked ubiquitination. The details of how specific DUBs regulates the activities of the TLRs and RLRs signaling molecules are described in the text.

The RLR family mainly consists of two members, RIG-I and MDA5, both caspase activation and recruitment domains (CARDs) containing cytosolic PRRs found in most cell types. RIG-I has the ability to preferentially recognize the viral 5′ triphosphorylated RNA in the cytosol of the infected cells [24,25]. Studies with RIG-I knockout cells prove the importance of RIG-I to detect influenza viruses, arenaviruses, and vesicular stomatitis virus (VSV). Accumulating evidence shows that during viral infection, K63-linked ubiquitination by the E3 ligase tripartite motif containing 25 (TRIM25, also called estrogen-responsive finger protein) and Riplet (also called RING-finger protein [RNF135] or REUL) facilitates the association of RIG-I and mitochondrial antiviral-signaling protein (MAVS) promoting type I IFN induction. However, recent studies have demonstrated that the deletion of TRIM25 in mouse and human cell lines does not affect immune response against influenza A virus, influenza B virus, or Sendai virus (SeV). Riplet is requisite for RIG-I-mediated IFN response during viral infection [26], whereas MDA5 activates IRF3 by interacting with unanchored K63-linked ubiquitin chains [27]. Upon virus recognition, Riplet-mediated K63-linked polyubiquitination is essential for the activation of RIG-I and for its association to TRIM25 [28]. Caspase-12 helps recruit TRIM25 and catalyzes the K63-linked ubiquitination of RIG-I, which leads to RIG-I oligomerization and interaction with MAVS [27,29]. MAVS modulates nuclear factor-κB (NF-κB) activity via tumor necrosis factor receptor-associated factor (TRAF) 6, an inhibitor of nuclear factor κB kinase (IKK) complex (IKK α/β/γ) activation and production of proinflammatory cytokines [30]. On the other hand, the autoubiquitination of TRAF3, which interacts with MAVS, induces the recruitment of Tank-binding kinase 1 (TBK1) and IKKε, and subsequently promotes the phosphorylation of IFN regulatory factor (IRF) 3 and IRF7. Phosphorylated IRF3 and IRF7 translocate to the nucleus and trigger type I IFN production (Figure 1) [31]. Subsequent studies demonstrated that certain E3 ligases like TRIM4 and MEX3C increase the type I IFN induction following viral infection [32,33]. Furthermore, the overexpression of TRIM44 enhanced the antiviral response by stabilizing MAVS and enhancing IFN induction [34].

### 2.2. K48-Linked Ubiquitination: RIG-I Degradation

The K48-linked ubiquitination of RIG-I by multiple E3 ligases leads to proteasomal degradation. E3 ligases, namely the C-terminus of Hsc70 interacting protein (CHIP), RNF122, and RNF125 mediate the proteasomal degradation of RIG-I by conjugating them with K48-linked ubiquitin chains inhibiting the antiviral innate response [35,36]. Another E3 ligase, c-cbl, is recruited by sialic acid binding Ig-like lectin G (Siglec-G) to facilitate the K48-linked ubiquitination of RIG-I [37]. TRIM40 promotes the K27- and K48-polyubiquitination of RIG-I, targeting it for degradation [38]. MAVS is ubiquitinated by the poly(C)-binding protein 2-atrophin 1-interacting protein 4 (PCBP2-AIP4) complex or by RNF5 for its degradation via the ubiquitin proteasome pathway. RNF catalyzes the K48-linked ubiquitination of stimulator of IFN genes (STING), leading to the degradation of STING and decreased immune response. However, RNF26 inhibits STING degradation by catalyzing its K11-linked ubiquitination, thus helping in creating an antiviral environment by the synthesis of type-I IFNs. E3 ligases TRIM32 and TRIM56 target STING for K63-linked ubiquitination, which is essential for the downstream signaling cascade during viral infection. STING also undergoes K27-linked ubiquitination by insulin-induced gene 1-autocrine motility factor receptor (INSIG1-AMFR), which promotes TBK1 recruitment to activate IRF3. Linear ubiquitin assembly complex (LUBAC), which consists of HOIL-1L, SHARPIN, and HOIP, competes with TRIM25 for binding to RIG-I. LUBAC also promotes K48-linked polyubiquitination to modify TRIM25, leading to its proteasomal degradation [13]. Lys63-linked polyubiquitination is a pivotal step for the activation of RIG-I, which leads to the induction of type I IFN and inflammatory cytokines by activating NF-κB and IRF3 transcription factors [39]. Ubiquitination can be reversed by the action of DUBs, indicating that DUBs negatively regulate antiviral response in macrophages.

### 2.3. Deubiquitination: Negative Regulators of RIG-I 

#### 2.3.1. CYLD

Cylindromatosis (called CYLD or ubiquitin carboxyl-terminal hydrolase), which is a deubiquitinating enzyme that removes K63-linked ubiquitin chains, also negatively regulates antiviral response [31]. CYLD physically interacts with both RIG-I and MAVS and preferentially inhibits the ubiquitination of RIG-I [40] along with TBK1 and IKKε, upregulating several IFN stimulating genes. Due to CYLD overexpression, IFN-β expression decreases, whereas the replication of SeV increases [41]. However, in the VSV model that specifically stimulates the RIG-I pathway, CYLD is essential for antiviral host defense. About 60% of CYLD^+/+^ mice survived viral infection, whereas CYLD^-/-^ mice were more sensitive, and only about 20% survived under the same conditions. These findings contrasted with prior reports; this might be because of feedback inhibition, wherein an excess of interferon stimulating genes (ISG) synthesis inhibits type I IFN synthesis to control the antiviral response. Nonetheless, CYLD plays a crucial role in antiviral innate response [42]. CYLD also facilitates the deubiquitination of multiple signaling molecules, such as members of the TRAF family, IKKγ, IKKε, TBK1, TAK1-binding protein 1 (TAK1), RIP1, and RIG [43,44]. Recent findings demonstrated that adaptor proteins are also involved in the function of CYLD. For example, the adaptor protein p62, which is required for CYLD binding to TRAF6, regulates the DUB activity of CYLD by promoting CYLD ubiquitination [45].

#### 2.3.2. A20

TBK1 is activated by the E3 ligases mind bomb 1 and 2 (MIB1 and MIB2), which mediated K63-linked ubiquitination via MAVS. Upon activation, these kinases phosphorylate the transcription factors IRF3 and IRF7, which subsequently leads to the production of IFN by binding to the enhancer/promoter region of the IFN β gene [46]. However, A20 negatively regulates IRF3 activation by removing K63-linked polyubiquitin chains from TBK1. Therefore, A20 might restrict the antiviral signaling response. A20 has both E3 ligase and deubiquitinase activity. According to a study of A20 along with its interacting protein, Tax1-binding protein 1 (TAX1BP1), antagonize the K63-linked polyubiquitination of TBK1 and IKKi, terminating the antiviral signaling independent of its DUB function. It was also observed that A20 and TAX1BP disrupt the interaction of E3 ligase TRAF3 and its substrate TBK1/IKKi. Likewise, A20, with its DUB-dependent function, disrupts the TRAF6-mediated K63-linked polyubiquitination of IRF7, blocking the IFN production [47]. 

#### 2.3.3. USP21

USP21 is a highly active and conserved deubiquitinase, which cleaves the K63-linked ubiquitin chain efficiently by inhibiting both TRIM25 and RNF135 [48,49,50]. Interestingly, the genetic deletion of USP21 in macrophages enhances IRF3 activation, IFN production, and antiviral response. The comparison between the deubiquitinase activities and binding affinities to RIG-I-CARD of USP21, A20, and CYLD indicate that USP21 is a bona fide RIG-I deubiquitinase, since among these three DUBs, USP21 is the only one that has the potential to inhibit RIG-I-CARD polyubiquitination both in vivo and in vitro and co-immunoprecipitates with RIG-I-CARD [51]. Overexpression studies in HEK293T cells revealed that USP21 might also bind and deubiquitinate MDA5 by removing K63-linked polyubiquitination, thus inhibiting IFN-β, NF-κB, and IFN-stimulated response element (ISRE) reporter activities. MDA5-mediated antiviral responses are thus inhibited by deubiquitinase USP21 [52].

#### 2.3.4. USP3

USP3 also negatively regulates IFN signaling by interacting with RIG-I and MDA5, but not with other downstream signaling proteins like MAVS, TBK1, IKKi, IRF3, TRAF3, or TRAF6. The interaction of USP3 with RIG-I and MDA5 is strictly based upon ligand stimulation. Upon viral infection, USP3 directly interacts with the CARD of RIG-I and cleaves the K63-linked polyUb chains, but not the K48-linked polyUb chain, thus converting RIG-I to its inactive form [53]. The ectopic expression of USP3 in HEK293T cells that are treated with low molecular weight poly (I:C) and THP-1 cells infected with VSV attenuated the IFN-β promoter activity. Conversely, the silencing of USP3 enhanced the phosphorylation of IRF3 and IFN-β protein expression under similar conditions. USP3 may bind to MDA5 via unanchored polyUb chains and then deubiquitinate it [53]. 

#### 2.3.5. USP15

USP15 has been demonstrated to promote RIG-I-mediated antiviral signaling by binding to N-terminal CARD. USP15 was originally reported to maintain TRIM25 in an inactive state by cleavage of the K48-linked ubiquitin chains, thus allowing the TRIM25-dependent activation of RIG-I [54]. However, as reported in a recent study, USP15 functions as the deubiquitinase of RIG-I that removes K63-linked polyUb chains. USP15 specifically deubiquitinates RIG-I by direct physical association and does not interact with other signaling mediators like interferon-beta promoter stimulator 1 (IPS-1), TRAF3, and TBK1 in HEK293 cells. Interestingly, USP15 competes with IPS-1 to bind to RIG-1, limiting IPS-1 activation and IFN1 production. The knockdown of USP15 increases RIG-I K63-linked polyubiquitination, enhancing the IFN production, whereas the overexpression of USP15 decreases the type I IFN by blocking the transcription of IFN-β in HEK293T infected with SeV. Thus, USP15 acts as a negative feedback molecule that is able to attenuate the antiviral state by modulating RIG-I [55].

#### 2.3.6. USP25

USP25 has an ability to cleave both K48- and K63-linked polyubiquitination chains. USP25 facilitates the deubiquitination of critical signaling molecules of the IFN signaling pathway, such as RIG-I, TRAF3, and TRAF6. USP25 negatively regulates the SeV-induced activation of ISRE in HEK293 cells. USP25 also inhibits the activation and phosphorylation of IRF3 and NF-κB [56]. Although direct contact has not been established between USP25 and RIG-I, USP25 may deubiquitinate K63-linked polyUb chains. The overexpression of USP25 downregulated IFN-β promoter activity and IFN-β expression in SeV-infected HEK293T cells, and the silencing of USP25 by siRNA enhanced the IFN-β promoter activity [57]. In contrast, Lin et al. suggested that USP25 is a positive regulator of antiviral response, due to its stabilization effect on TRAF3 and TRAF6 upon viral induction triggering type I IFN induction [58]. IFN stimulates ISRE-containing promoters, resulting in the expression of ISGs to evoke antiviral activity [56]. The promoter of USP25 possesses multiple ISRE and κB binding sites that are recognized by IRF7 [58]. However, the interaction of USP25 with MDA5 has not been established and might be an important aspect to explore.

#### 2.3.7. USP14

USP14 is another negative regulator of the RIG-I-mediated antiviral response that deubiquitinates K63-linked polyUb chains from RIG-I. The siRNA knockdown of USP14 in mouse peritoneal macrophages resulted in higher IFN-β production and higher phosphorylation of IRF3 and TAK1 when compared to VSV infected cells. These data suggest that USP14 deficiency elevates IFN-β production and enhances the antiviral response exhibited by macrophages [59].

#### 2.3.8. USP18

USP18 plays a role in macrophage-mediated antiviral responses by recruiting USP20 to de-conjugate K48-linked ubiquitin chains from STING as a mediator of innate antiviral signaling. Immunoprecipitation revealed that both USP18 and USP20 interacted with the N-terminal region of the STING protein. As a functional consequence, after DNA virus infection, the deubiquitination of STING leads to the promotion of stability of STING, proinflammatory cytokines, and type-I IFN expression [60].

### 2.4. Positive Regulators of RIG-I 

#### USP4 and USP17

USP4 has the ability to cleave K48-linked polyUb from RIG-I, which leads to protein stabilization. Thus, USP4 positively regulates IFN-β signaling by interacting with the N-terminal domain of RIG-I, subsequently leading to the inhibition of RIG-I degradation. Another DUB, USP17, is also known to deubiquitinate RIG-I by removing K48-linked polyUb from RIG-I. Upon SeV infection, the knockdown of USP17 inhibits the synthesis of type I IFN in RIG-I mediated signaling [61,62].

## 3. Ubiquitination and Deubiquitination in TLR Signaling

### 3.1. Ubiquitination of TLR Signaling Molecules: TRAF3, TRAF6, TBK1, RIP1, TAK1

The germline-encoded TLR family consists of 10 members in humans that are expressed by innate immune cells such as monocytes, dendritic cells, and macrophages. They recognize a wide range of bacterial and viral structures and initiate the signaling cascade by binding to either of its adaptor molecules: myeloid differentiation primary response (MyD) 88 or Toll/Interleukin-1 receptor (TIR)-domain-containing adapter-inducing interferon-β (TRIF) [63,64]. MyD88 forms a complex by recruiting IL-1R-associated serine/threonine kinases (IRAK1, IRAK2, and IRAK4) via its death domain, which then mediates the activation of TRAF-6, an E3 ligase. TRAF-6 polyubiquitinates itself and generates K63-linked polyubiquitin chains that bind other proteins like IRAK1, IRAK4, and MyD88. In addition, E3 ligase Pellino-1 generates K63-linked polyubiquitin chains. These anchored or unanchored polyubiquitin chains serve as binding sites for the ubiquitin-binding domain of TAB1/2, forming a TAK1-TAB1-TAB2 kinase complex and activating IKK to promote MAPK signaling, NF-κB activation, and TNF receptor-1 (TNFR1)-mediated proinflammatory cytokines production. On the other hand, TRIF binds to TLR3 and TLR4 through its TIR domain and recruits RIPK1 along with E3 ligases TRAF6 and cIAP1/2. As mentioned above, these E3 ligases activate TAK1 kinase by producing K63-linked polyubiquitin chains, thereby activating IKK and MAPK. Protein levels of MyD88 are tightly regulated by TGF-β-induced, K48-linked ubiquitination by the E3 ligase Smurf1/2 and neuregulin receptor degradation protein 1 (Nrdp1) [65]. Nrdp1 activates IRF3 by attaching K63-linked polyubiquitination to TBK1 [66,67]. TLR can initiate the degradation of its own receptors with the help of an E3 ligase, Traid3A. Modification with K48-linked ubiquitination to TLR3, TLR4, TLR5, and TLR9, but not TLR2, leads to their degradation. Triad3A may also regulate the ubiquitination and degradation of receptor-interacting protein 1 (RIP1) and TRAF3 [68,69]. 

### 3.2. Deubiquitination of TLR Signaling Molecules

#### 3.2.1. USP2/USP2a

USP2a, a 69-kDa splice variant of USP2, also known as USP9 and UBP41, negatively regulates NF-κB activation and proinflammatory cytokine production by interacting with TRAF6. When HEK293 cells were induced by IL-1β and SeV, overexpression of USP2a interacted with TAK1 and TRAF6 to inhibit NF-κB activation, whereas USP2a-deficient cells enhanced the phosphorylation of IKKα/β and NF-κB activation. USP2a promotes the removal of K63 polyUb from TRAF6 and not K48-linked ubiquitin, thus negatively regulating NF-κB signaling in HEK293 and HCT116 [70]. However, in a recent study, USP2a positively regulated TRAF6-NF-κB signaling in T lymphocytes. This variance may be due to the use of different cell lines in the study. In LPS-stimulated macrophage-like cell line J774.1, the knockdown of USP2 promoted cytokine production, whereas the ectopic expression of USP2 repressed cytokine production. USP2 has the ability to control about 20% of 104 cytokines in HL-60 macrophages by deubiquitinating octamer-binding transcription factor-1 (Oct-1) transcription factors [71].

#### 3.2.2. USP4

USP4 negatively regulates TNF*α*-induced, K63-linked TAK1 polyubiquitination and TAK1-mediated IKK/NF-κB activation [72]. USP4 is reported to remove K63-ployUb from TRAF6 and TAK1, activating NF-κB to produce proinflammatory cytokines [73]. USP4 deubiquitinates TRAF2 and TRAF6, but not TRAF3, and it also rescues IκBα from degradation in TNF*α*-induced HEK cells [73]. More recently, Xu et al. demonstrated that upon enterovirus 71 (EV71) infections, USP4 expression was repressed, whereas the overexpression of USP4 suppressed EV71 replication. Hence, USP4 positively regulates NF-κB signaling by deubiquitinating K48-ubiquitin chains of TRAF6 in EV71 infection [74].

#### 3.2.3. USP7

USP7 was first identified as a viral binding protein. USP7 interacts with proteins that are important for viral growth and replication like ICP0 (Vmw 110), a herpes simplex virus immediate-early gene [75,76], and Epstein-Barr nuclear antigen-1 (EBNA1), another herpes virus protein [77,78]. Interestingly, USP7 shows opposing functions depending on its cellular localization. In the nucleus, USP7 increases NF-κB stability by regulating NF-κb transcriptional activity [79]. However, in the cytosol, it acts as a negative regulator of the NF-κB via NEMO and TRAF6 deubiquitination, which causes NF-κB retention in the cytosol [80]. TLR- and TNFR-induced inflammatory gene expression is regulated by USP7, which was confirmed when the LPS-induced expression of IL-6, TNFα, and IL-23p19 was inhibited by HBX41,108, a USP7 inhibitor in murine macrophages. However, USP7 deubiquitinates the NF-κB p65 subunit, which is an important regulator of inflammation, but the USP7 inhibitor targets all the substrates of USP7, not only p65. Therefore, an inhibitor targeting specifically the NF-κB and p65 interaction would be a potential future therapeutic [79]. All in all, DUB inhibitors are potential drugs that target IL-1-related inflammatory disease.

#### 3.2.4. USP10

The NF-κB pathway is also activated due to DNA damage to handle genotoxic stress responses. In this case, USP10 forms a complex with monocyte chemoattractant protein-induced protein (MCPIP) and TANK to essentially deubiquitinate TRAF6, repressing the DNA damage mediated-NF-κB activation. USP10′s association with MCPIP was able to deubiquitinate the linear-polyUb chain from NEMO when exposed to genotoxic stimuli [81,82].

#### 3.2.5. USP18

An IFN inducible gene, USP18 (also known as UBP43) can remove K63-polyUb and is upregulated in response to TLR ligands in human macrophages. USP18 deubiquitinates TAK1 in a protease-dependent manner in HEK293 cells. Moreover, USP18 masks the ubiquitin sites of NEMO by binding to its ubiquitin-binding ABIN and NEMO (UBAN) sites, protecting them from further K63-ubiquitination. Hence, USP18 is a negative regulator of TLR- induced NF-κB activation in the innate immune response [83]. 

#### 3.2.6. USP25

During TLR4-mediated signaling, TRAF3 is regulated by USP25, which is known as an essential endotoxin tolerance (ET) regulator in macrophages as well as functioning in protein degradation associated with ER and in cell migration and invasion [57,84]. Deficiency of USP25 in KCs leads to the LPS-induced ubiquitination of TRAF3, which attenuates anti-inflammatory cytokine production and upregulates the expression of proinflammatory cytokines [85]. Therefore, the interaction between USP25 and TRAF3 eliminates K48-linked polyubiquitin chains from TRAF3 and causes ET. These findings indicate that understanding the roles and mechanisms of DUBs leading to ET may provide an improved treatment for sepsis.

#### 3.2.7. USP12

As described in previous sections, the bacterial endotoxin LPS, which stimulates the NF-κB pathway, causes macrophage-mediated inflammatory responses. One of these inflammatory response regulators is USP12. Most of the deubiquitinases, including A20 and CYLD, can inhibit NF-κB activation [86], while USP12 positively regulates the LPS signal [87]. LPS binds to TLR4, which is a transmembrane protein on macrophages, triggering signal transduction through the NF-κB pathway. Mechanistic analysis demonstrates that USP12 dephosphorylates IκBα, which is a well-characterized inhibitor of NF-κB nuclear translocation, inducing proinflammatory responses in macrophages. There are several binding sites for different transcription factors such as Sp1, NF-κB, STAT1, and CREB in the upstream region of USP12 gene. Over-expression of Sp1 and p65 upregulates the promoter activity of USP12. Furthermore, LPS-induced macrophage activity is enhanced by Sp1 levels at earlier time points. In addition, the ablation of USP12 expression in mouse macrophages by siRNA reduces LPS-induced iNOS and IL-6 expression. Other studies have shown that USP12 suppresses the PH domain and leucine-rich repeat protein phosphatases, which restrains the activation of macrophages through the dephosphorylation of transcription factor STAT1 [87]. These findings suggest that USP12 can be a therapeutic target in patients with hyper-activation of macrophages.

#### 3.2.8. A20

A20 interferes with TLR-mediated IFN and NF-κB activation. The membrane-bound TLRs activate an antiviral signaling cascade against viral invasion. TLR3 and TLR4 recognize the double-stranded RNA and bacterial LPS, triggering the production of inflammatory cytokines and type I IFN. A20 targets TRAF6-mediated antiviral signaling in negative feedback regulation analogous to IL-1R and TLR4 signaling inhibition [88]. During intestinal inflammation, A20 negatively regulates TLR5 signaling in intestinal epithelial cells [89]. A20 possess anti-inflammatory function and, hence, mice deficient in A20 (A20^−/−^) show inflammation in various organs, cachexia, and early death [90]. Additionally, A20 is essential for the termination of the TLR-induced activity of NF-κB and proinflammatory genes in macrophages [90,91].

#### 3.2.9. CYLD

CYLD does not have an essential role in NF-κB activation by TLR ligands in macrophages, since the loss of CYLD has negligible effects on it [43]. The function of CYLD is likely the same as that of A20, since A20 is expressed in both a basal and inducible manner by macrophages [90]. However, CYLD does not have a compensatory role for A20, because of the attribution of TLR and TNFR signals to the inactivation of CYLD in stimulated cells by CYLD phosphorylation [31]. MyD88, an important molecule in TLR and IL1-receptor mediated signaling, undergoes K63-linked polyubiquitination, which is negatively regulated by CYLD. Studies on MyD88^−/−^ CYLD^−/−^ mice confirmed that CYLD deubiquitinates the K63-polyubiquitination of MyD88; however, it is unclear whether CYLD directly inhibits MyD88 signaling [92]. CYLD also dissociates K63-ubiquitination from RIPK1, an essential molecule for necroptosis. To protect against TNF-induced necroptosis, TLR4 activates caspase-8 to cleave and remove CYLD in macrophages [93].

#### 3.2.10. MYSM1

The H2A deubiquitinase myb-like SWIRM and MPN domains 1 (MYSM1, also known as 2A-DUB or KIAA1915) is a negative regulator of proinflammatory cytokines and type I IFN response. The two domains of MYSM1 interact with TRAF3 and TRAF6 to terminate the PRR pathways. The SWIRM motif interacts with TRAF3 and TRAF6, and the C-terminal of the metalloproteinase domain deubiquitinates K63-polyUb chains. In MYSM1-deficient mice, hyper-inflammation and increased viral clearance was observed along with susceptibility to septic shock [94].

## 4. Ubiquitination in NLR Signaling

### 4.1. Ubiquitination of NLRP3 and RIP2

**Figure 2:**
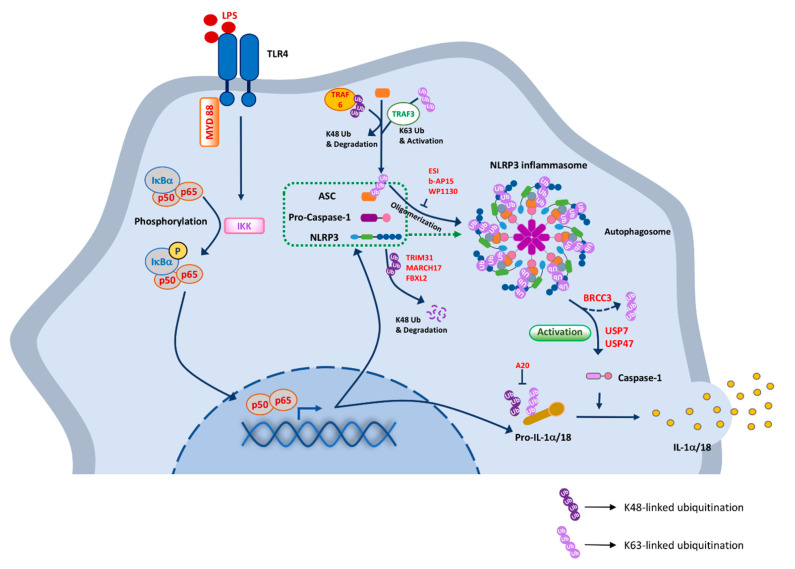
Schematic diagram of the mechanism of NLRP3 inflammasome activation by ubiquitination in macrophages. Inflammasome requires a priming signal, such as LPS, that activates the NF-κB subunits (p50 and p65), leading to upregulation of pro-IL-1β/IL-18 and NLRP3 genes in macrophages. Activation signals, such as ATP and bacterial toxins, increase potassium influx, which recruits ASC and Caspase-1 to assemble the active NLRP3 inflammasome complex. Caspase-1 generated by active NLRP3 inflammasome cleaves and secretes proinflammatory cytokines such as IL-1β and IL-18. Dark purple circles indicated K48-linked ubiquitination and light purple circles indicate K63-linked ubiquitination. The details of how specific DUBs and DUB inhibitors regulate the activities of the NLRP3 inflammasome signaling molecules are described in the text.

The NLR family includes cytoplasmic PRRs involved in the formation and activation of inflammasomes, the secretion of inflammatory cytokines, and also some non-inflammasome functions. NLRs can recognize PAMPs and DAMPs and regulate inflammasome and non-inflammasome function. There are more than 23 members in NLR family; for example, the well-characterized member NLRP3 is involved in inflammasome function. NLRP3, with the help of its adaptor protein ASC, binds to caspase-1, thus activating it. Activated caspase 1 mediates the activation and release of pro-IL-1β and pro-IL-18 (Figure 2). Certain E3 ligases are known to regulate NLRP3 activation by targeting NLRP3 or its components, ASC and caspase 1. TRIM31 negatively regulates NLRP3 activation by conjugating it with K-48 polyubiquitination, resulting in the proteasomal degradation of NLRP3 [95]. Another E3 ligase, SCF-FBXL2, ubiquitinates and degrades NLRP3. However, after LPS stimulation, E3 ligase F-box protein 3 (FBXO3) levels increase, resulting in the increased degradation of SCF-FBXL2. This results in decreased interaction between FBXL2 and NLRP3, thus increasing its availability for NLRP3-mediated inflammasome activation [96]. To avoid NLRP3-mediated inflammasome activation, the NLRP3 is paused in its inactive state by E3 ligase Cullin1. Independent of degradation, Cullin1 maintains NLRP3 in an inactive state, and upon an activation signal, Cullin1 detaches from NLRP3, allowing it to form an active inflammasome complex [97]. Other E3 ligases, such as Ariadne homolog 2 (ARIH2), Parkin, or membrane-associated RING-CH-type finger 7 (MARCH7), have been identified to negatively regulate NLRP3 [98]. NLRP3 is activated by K63-specific deubiquitination by BRCA1/BRCA2-containing complex 3 (BRCC3) [99,100]. 

E3 ligases can also promote inflammasome activation, like Pellino2, TRAF6, and TRIM33. Pellino2 is found to ubiquitinate NLRP3 for inflammasome activation and also IRAK1 to limit its ability to inhibit NLRP3 [101]. In a recent report, it was demonstrated that TRAF6-deficient cells had impaired NLRP3 inflammasome activation, but the mechanism behind this is not understood completely. TRAF6 is also known to ubiquitinate and degrade ASC by autophagy in macrophages [102]. LUBAC, consisting of HOIP, HOIL, and SHARPIN, conjugates linear polyubiquitin chains to ASC. In this line of work, LUBAC is essential for NLRP3 inflammasome activation. HOIL-1- and SHARPIN-deficient macrophages were shown to have impaired NLRP3 inflammasomes [103,104].

The bacterial peptidoglycan stimulates NOD1 and NOD2 and it binds to RIP2, facilitating its K63-linked polyubiquitination and thereby activating TAK1 and IKK. RIP2 ubiquitination is regulated by several E3 ligases, such as Peli3, XIAP, cIAP1, cIAP2, and TRAF family members like TRAF2, TRAF5, TRAF6, and ITCH [21]. NLRs also regulate TLR signal transduction and inhibit the inflammatory response. NLRP12 is associated with NIK and TRAF3 and negatively regulates the NF-κB pathway. TRAF3 recruits NIK to E3 ligase cIAP, which ubiquitinates and degrades NIK [105]. Another NLR member, NLRX1, and TRAF6 negatively regulate NF-κB activation by the K63-linked polyubiquitination of NLRX1, which is then associated with the IKK complex inhibiting NF-κB activation [106]. These studies highlight the importance of the different cellular environments for the activity and function of E3 ligases.

### 4.2. Deubiquitination of NLR Signaling Molecules

#### 4.2.1. A20

A20 is the only DUB that negatively regulates the inflammasome formation. Duong et al. demonstrated that A20-deficient macrophages facilitate RIPK3-dependent spontaneous NLRP3 inflammasome response upon LPS induction. A20 associates with caspase-1 and pro-IL-1β and forms a complex with caspase-8, RIPK1, and RIPK3 that is modified with K63-linked and unanchored polyUb [107]. Caspase-1 is a crucial proinflammatory caspase that helps in release of proinflammatory cytokines pro-IL-1β and pro-IL-18. A20-deficient macrophages promoted NLRP3 inflammasome activation along with caspase-1 and the release of IL-1β [108].

#### 4.2.2. BRCC3

BRCC3 or BRCC36 are JAMM domain-containing Zn^2+^ metalloprotease DUBs. BRCC3 is known to regulate the deubiquitination of NLRP3 in the cytosol and is a part of the BRCC36 isopeptidase complex (BRISC) that specifically cleaves K63-polyUb from NLRP3 and is essential for NLRP3 activation [99]. Another subunit of the BRISC complex, ABRO1, is important for NLRP3 activation. ABRO1 binds to NLRP3 and recruits BRISC to mediate the deubiquitination of NLRP3 in macrophages [100].

#### 4.2.3. USP7 and USP47

The catalytic site of USP7 is similar to that of USP47, which is its closest related DUB. Inhibition of USP7 and USP47 by P22077 plays its role by preventing ASC speck formation, leading to impaired inflammasome formation in macrophages and ubiquitination status of NLRP3. The roles of USP7 and USP47 in NLRP3-mediated inflammasome activation were confirmed using CRISPR/Cas9 technology in the macrophage cell line THP-1. This study showed that inflammasome activation is reduced when both *USP7* and *USP47* are downregulated [109]. Upon inflammasome activation signals, the enzymatic activity of USP7 and USP47 increases in macrophages. However, there is no evidence of direct interaction between USP7, USP47, and NLRP3 [21]. On the other hand, USP7 is known to deubiquitinate K48-linked polyUb from the NF-κB-p65 subunit of the NF-κB complex, thus stabilizing it and promoting its occupancy in NF-κB-targeted promoters [79]. Therefore, USP7′s deubiquitination of NF-κB enhances pro-cytokine production when induced by the TLR signaling pathway. The DUBs implicated in the regulation of NF-κB pathway and inflammasome pathway have been summarized in Table 1.

## 5. DUB Inhibitors

UPS is associated with multiple pathologies including cancers, inflammation, thus suggesting its great potential as a pharmacological target for drug development. Recently, targeting the proteasome has proven a successful clinical therapy, that has come in the form of FDA approval of a few proteasome inhibiting molecules such as bortezomib [114], carfilzomib [115], oprozomib (ONX0912) [116], and ixazomib [117]. Proteasomal inhibitors have also been recognized for its anti-inflammatory properties, however, it has only proven success for the treatment of multiple myeloma, due to its toxic side effects. Additionally, the ubiquitin-activating enzymes hold most potential because of the presence of a well-defined activity pocket. However, these enzymes lack specificity as they can influence thousands of protein that are regulated by the UPS. Only a few of E1-enzyme inhibitors such as, MLN7243 (NCT03816319), MLN4924 [118,119], and Compound 4b [120] have thus entered clinical trials for leukemia and epilepsy. Development of E3 ligase inhibitors have proven to be more efficient, specific and less toxic in cancer development [121]. Numerous E3 ligases have been identified in regulating the macrophages-mediated signaling, however, development of E3 ligase inhibitor is still in its infancy. Thus a more judicious approach is needed for developing molecules targeting E3 ligases involved in macrophage signaling. In contrast, DUB inhibitors have gained a very large amount of success in the past 10–15 years with the preclinical development of candidate compounds that are highly selective. Thus the accomplishment of DUB inhibitors in the clinical or late preclinical development and its well defined active site makes them a simpler therapeutic target. Thus, DUB inhibitors which are involved in macrophage-mediated signaling pathways are discussed in this section.

Many viruses have been reported to evolutionarily acquire different strategies to utilize the ubiquitin pathway for their own benefits [122]. During infections, the intracellular pathogens and viruses have the capacity to manipulate the ubiquitin cycle to their advantage by encoding DUBs [123]. USP14 is a proteasome-associated DUB that is reported to retard protein degradation with interaction with the cytoplasmic region of inositol-requiring enzyme 1 alpha, which is a critical mediator of the unfolded protein response [124]. USP14 has been identified as a negative regulator in antiviral responses by directly deubiquitinating K63-linked RIG-I [59]. Thus, modulation of viral infections could be achieved by understanding how to inhibit the biological activity of USP14 associated with the immune system. 

Several studies have demonstrated that the small molecule WP1130 acts as a partially selective DUB inhibitor [125]. It has been reported that WP1130 inhibits the activity of a set of DUBs including USP14 in Z138 mantle cell lymphoma cells [125], indicating that it may provide a unique therapeutic approach for an antitumor strategy. In addition, WP1130 contributes to the accumulation of ubiquitinated proteins and antibacterial effects against *Listeria monocytogenes* in murine macrophages [126]. More importantly, treatment with WP1130 in murine macrophages with viral infections resulted in significant reduction of the replication of murine norovirus 1 (MNV-1), encephalomyocarditis virus, Sindbis virus, and La Crosse virus [127]. However, the poor solubility and bioavailability of WP1130 limit its utility [127]. This obstacle is removed by identifying compound 9, which is designed based on WP1130 structure. In comparison to WP1130, compound 9 is more soluble and has an anti-infective activity potential at lower concentrations [128]. Therefore, compound 9 can be a potential drug against diverse microorganisms including *Listeria monocytogenes* and MNV-1. More recently, the 2-cyano-3-acrylamide (compound C6) has been identified as a more efficient DUB inhibitor with lower toxicity than compound 9 that promotes the inhibition of the intracellular replication of MNV-1 and *Listeria monocytogenes* in murine macrophages [129]. All these findings indicate that targeting USP14 with small molecules could be a potential therapeutic strategy for wide-spectrum antiviral therapies.

### 5.1. DUB Inhibitor (WP1130) for Bacterial Killing in Macrophages 

Generally, bacteria within the phagosome are compromised with antimicrobial effectors, such as iNOS, phagocyte NADPH oxidase (phox), and producers of NO and superoxide, which form highly reactive peroxynitrite through DNA mutagenesis and exert direct toxic effects that can reduce the viability of microbial generations and the survival of bacteria [130]. WP1130 catalyzed this process, altered the survival of bacteria within the phagosome, and induced bacterial killing more rapidly by the induction of iNOS localization to the bacterial phagosome by modestly inducing total iNOS activity while the overall cellular abundance of iNOS did not change [126]. This suggests the potential role of ubiquitin and DUBs in iNOS trafficking regulation [126].

### 5.2. DUB Inhibitors in Inflammasome Assembly

The activation of macrophages is initiated by inflammasome assembling. This process requires the adaptor protein ASC to bring the receptor and the zymogen pro-caspase-1 into proximity. Formation of the inflammasome occurs by assembling both NLRP3 and AIM2 receptors [131]. Accumulating evidence suggests that ubiquitination and autophagy are involved in regulating the formation and activation of inflammasomes. The formation and maturation of autophagosomes demand microtubule-associated protein1 light chain 3B (LC3B) [132]. Deficiency of LC3B in mouse peritoneal macrophages enhances the activation of caspase-1 and the secretion of IL-1β/18 [133]. The ubiquitination of ASC is targeted by autophagy and regulates inflammasome activity [134]. According to a recent study, DUB inhibitors, such as eeyarestatin I (ESI), b-AP15, and WP1130, inhibit ASC oligomerization and consequently caspase-1 activation (Figure 2) [135]. ESI, an ER-associated protein degradation inhibitor, inhibits protein translocation in the ER and induces an ER stress response [136]. ESI inhibits the expression of IL-1β by inhibiting deubiquitination processes [137]. In addition, b-AP15 is a small molecule targeting two DUBs, UCHL5 and USP14 [138]. ATP-induced IL-1 release from LPS-primed peritoneal macrophages is inhibited by pretreatment with b-AP15 [135]. Furthermore, the potential involvement of DUBs in IL-1 release was evaluated using another DUB inhibitor, WP1130. USP14, UCH37, USP5, and USP9x activities are directly blocked by WP1130 [125]. WP1130 also inhibits the activation of caspase-1 in response to NLRP3 inflammasome-activating stimuli [139]. Based on the literature demonstrating the function of DUB inhibitors that block the oligomerization of ASC, DUBs appear to regulate upstream of inflammasome assembly. Thus, there is the possibility that the accumulation of ubiquitinated inflammasome components and the upregulation of the consequent degradation by autophagy in the macrophage without altering the autophagy levels are caused by DUB inhibitors [135]. 

## 6. Conclusions

The E3 ligases and DUBs are essential proteins within receptor signaling complexes. DUBs have been a fascinating frontier for research addressing their relevance in biological processes and cellular communication pathways, but their involvement in innate immune defense is less understood. Immune response requires tight regulation and control. Uncontrolled immune response leads to immunodeficiency, damage, and chronic inflammatory diseases [140]. Recent studies have provided important insights into how protein ubiquitination modulates not only the activation of antimicrobial response and inflammasome assembly, but also the signal transduction of critical cellular proteins [12,67]. Hence, ubiquitination has proved to be a crucial mechanism to control the host innate and adaptive immune response. Depending on the type of polyubiquitin linkage, the immune response can be propagated or terminated resulting in the degradation or activation of regulatory proteins. Indeed, ubiquitination is important for fine tuning the PRR signaling, regulation of NF-κB signaling, T cell activation and differentiation [141], and dendritic cell functioning and maturation [142,143,144]. Perturbation of ubiquitin homeostasis leads to immunological disorders like autoimmune and inflammatory diseases, such as encephalomyelitis, arthritis, and diabetes [141,145]. Hence, striking a balance between the function of E3 ligase, to add ubiquitin, and that of DUBs, to remove ubiquitin, plays a crucial role in maintaining cellular equilibrium.

In response to the importance of ubiquitination, pathogens have evolved machinery to exploit the ubiquitination system to promote infection by mimicking the host E3 ligases and DUBs [146,147]. Leveraging this characteristic, therapeutic inhibitors are being developed to alter the outcomes of intracellular infections. Small molecule inhibitors of DUBs present great potential for prevention and possibly can be a first line of therapy against innate immune disorders. These inhibitors interfere with the interaction between a DUB and the substrate protein inhibiting its enzyme activity. Intensive research has been performed where DUB inhibitors were proven to be powerful regulators in diseases like cancer [148], neurological disorders [149], infectious diseases, and autoimmune and inflammatory disorders [150]. DUBs have a wide range of functions and can regulate several cellular processes. In this review, we discussed the role of DUBs in the innate immune response led by the macrophages. Although a number of DUBs regulate the immune response, there are several more to be discovered. 

## Figures and Tables

**Table 1:** DUBs regulating RLR, TLR, and NLR signaling and their mechanism.

Pathway	DUBs	Target	Mechanism	Reference
**RLR**	CYLD	RIG-I, MAVS, TBK1, IKKε, TRAF2, TRAF3, TRAF6	Deubiquitinates K63-Ub chain	[41,44,90,110,111]
	A20	TBK1, IKKi, TRAF6, IRF7	Deubiquitinates K63-Ub chain	[47,90,112]
	USP21	RIG-I, MDA5	Deubiquitinates K63-Ub chain, Inhibits TRIM25 and RNF135	[51,52]
	USP3	RIG-I, MDA5	Deubiquitinates K63-Ub chain	[53]
	USP15	RIG-I	Deubiquitinates K63-Ub chain	[54,55]
	USP25	RIG-I, TRAF2, TRAF3, TRAF6	Deubiquitinates K63-Ub chain from RIG-I and TRAF6, Deubiquitinates K48-Ub chain from TRAF3	[57,58]
	USP14	RIG-I	Deubiquitinates K63-Ub chain	[59]
	USP18	TAK1, NEMO	Recruits USP20 to deconjugate K48-Ub chain from STING	[60]
	USP4	RIG-I	Deubiquitinates K63-Ub chain	[61,62]
	USP47	RIG-I	Deubiquitinates K63-Ub chain	[62]
**TLR**	USP2/USP2a	TRAF6	Deubiquitinates K63-Ub chain	[70,71]
	USP4	TAK1, TRAF2, TRAF6	Deubiquitinates K63-Ub from TAK1 and TRAF6, Deubiquitinates K48-Ub from TRAF6, rescues IκBα from degradation	[72,73,74]
	USP7	NF-*κ*B, NEMO, TRAF5	Deubiquitination	[75,76,77,78,79,80]
	USP10	NEMO, TRAF6	Deubiquitinates K63-Ub from TRAF6, Decrease M1-linked Ub from NEMO	[81,82]
	USP18	TAK1, NEMO	Deubiquitinates K63-Ub from TAK1, masks ubiquitin sites of NEMO	[83,113]
	USP25	TRAF3	Deubiquitinates K48-Ub	[57,84,85]
	USP12	NF-*κ*B	promotes LPS induced signaling through the dephosphorylation of IκBα.	[86,87]
	A20	TRAF6, RIPK1, NEMO	Deubiquitinates K63-Ub from RIPK1, attaches K48-Ub to RIPK1, inhibits ubiquitination of TRAF6, blocks NF-κB activation by binding to NEMO	[88,89,90,91]
	CYLD	MyD88, RIPK1, TRAF2, NEMO	Deubiquitinates K63-Ub from MyD88, RIPK1, TRAF2 and linear-Ub from NEMO	[43,92,93]
	MYSM1	TRAF3, TRAF6	Deubiquitinates K63-Ub	[94]
**NLR**	A20	RIPK2, RIPK3	Deubiquitinates RIPK2	[107,108]
	BRCC3	NLRP3	Deubiquitinates K63-Ub	[99,100]
	USP7	NF-*κ*B-p65, NLRP3	Deubiquitinates K48-Ub from NF-*κ*B-p65, regulates NLRP3 directly or indirectly	[21,79]
	USP47	NLRP3	Regulates NLRP3 directly or indirectly	[109]

## Abbreviations

PRR	Pattern recognition receptors
PAMPS	Pathogen-associated molecular patterns
DAMPS	Danger-associated molecular patterns
DUB	Deubiquitinating enzyme
KC	Kupffer cell
TLR	Toll-like receptors
RIG-I	Retinoic acid-inducible gene I
RLR	RIG-I- like receptors
MDA5	Melanoma-differentiation-associated gene 5
NOD	Nucleotide-binding oligomerization domain
NLR	NOD-like receptors
IFN	Interferons
IL	Interleukin
AIM2	Absent in melanoma 2
NLRP3	Nucleotide-binding oligomerization domain leucine-rich repeat and pyrin domain-containing protein 3
ASC	Apoptosis-associated speck-like protein containing a caspase activating and recruitment domain
CARD	Caspase activation and recruitment domains
VSV	Vesicular stomatitis virus
TRIM25	Tripartite motif containing 25
MAVS	Mitochondrial antiviral-signaling protein
NF-κB	Nuclear factor-κB
TRAF	Tumor necrosis factor receptor-associated factor
IKK	Inhibitor of nuclear factor κB kinase
TBK1	Tank-binding kinase 1
IRF	IFN regulatory factor
CHIP	C-terminus of Hsc70 interacting protein
RNF	RING-finger protein
STING	Stimulator of IFN genes
LUBAC	Linear ubiquitin assembly complex
TAX1BP1	Tax1-binding protein 1
ISRE	IFN-stimulated response element
MyD88	Myeloid differentiation primary response 88
TIR	Toll/Interleukin-1 receptor
TRIF	TIR-domain-containing adapter-inducing interferon-β
IRAK	IL-1R-associated serine/threonine kinases
TNFR1	TNF receptor-1
EV71	Enterovirus 71
MNV 1	Murine norovirus 1
FBXO3	F-box protein 3
BRCC3	BRCA1/BRCA2-containing complex 3
BRISC	BRCC36 isopeptidase complex
EMPs	Erythromyeloid progenitors
HSCs	Hematopoietic stem cells
IPS-1	Interferon-beta promoter stimulator 1
ISG	Interferon stimulating genes
